# Beyond clinical urgency: parental awareness, educational level, and non-urgent pediatric emergency department utilization

**DOI:** 10.3389/fped.2026.1890076

**Published:** 2026-07-22

**Authors:** Mustafa Ozgul, Bensu Ozec, Ali Boz, Ilknur Fidanci, Aslinur Coban, Ayman Altallawi, Zeynep Capar, Medine Aysin Tasar

**Affiliations:** 1 Department of Pediatric Emergency, University of Health Sciences Ankara Training and Research Hospital, Ankara, Türkiye; 2 Department of Pediatrics, University of Health Sciences, Ankara Training and Research Hospital, Ankara, Türkiye; 3 Medical Student, Faculty of Medicine, Yuksek Ihtisas University, Ankara, Türkiye; 4 Department of Nursing, University of Health Sciences, Ankara Training and Research Hospital, Ankara, Türkiye

**Keywords:** health care utilization, health literacy, parental awareness, pediatric emergency medicine, primary care utilization, triage

## Abstract

**Objective:**

Non-urgent visits to pediatric emergency departments (PEDs) constitute a persistent global problem that strains healthcare resources and may compromise care quality for critically ill patients. This study aimed to examine the socio-educational determinants of parental responsiveness to emergency-use informational messaging and to characterize the healthcare-seeking behaviors underlying low-acuity PED utilization at a tertiary-level center with an annual pediatric caseload of approximately 120,000 visits.

**Materials and Methods:**

In this prospective cross-sectional study, data were collected from parents via structured questionnaires administered during triage. Children triaged into the high-acuity Red or Orange Manchester Triage System (MTS) categories were excluded a *priori*, while Green and Blue categories were combined into a single lower-acuity group based on their comparable clinical management pathways. Multivariable logistic regression was used to identify independent predictors of an affirmative parental response to informational messaging on appropriate emergency department use.

**Results:**

A total of 453 parents were included in the study. Participants were predominantly female (68.2%), with a median parental age of 36.0 years, and 64.2% of visits were classified into the lower-acuity (Green/Blue) group. Agreement between parental urgency perception and MTS triage category was minimal (Cohen's *κ*=0.040, 95% CI −0.035 to 0.116; PABAK=−0.051), with no significant association between subjective urgency perception and objective triage classification (*p* = 0.298). Parental educational level was the strongest independent predictor of affirmative responsiveness to informational messaging, with each one-level increase associated with 1.45-fold higher odds (adjusted OR = 1.45; 95% CI: 1.14–1.86; *p* = 0.003). Across triage categories, the expectation of faster medical evaluation was the leading motivation for PED attendance (36.1%). Among lower-acuity presentations, 38.1% required laboratory or radiological investigations and 7.9% resulted in observation or inpatient admission, compared with 53.0% and 24.7%, respectively, for the whole cohort. When offered the alternative of after-hours outpatient services, 53.4% of parents indicated they would use them.

**Conclusions:**

These findings suggest that PED overcrowding is driven not solely by parental knowledge deficits but also by systemic barriers in primary care accessibility and parents' low risk tolerance. Effective interventions should therefore extend beyond public health education to restructure after-hours primary care as a credible and accessible alternative to emergency services.

## 1 Introduction

Approximately 40% to 80% of pediatric emergency department visits involve non-urgent clinical conditions that do not necessitate immediate medical intervention (1–3). Trends in healthcare utilization in Türkiye indicate that demand for emergency departments far exceeds system capacity. A significant portion of this visit burden consists of cases classified as low-priority according to medical triage standards that could appropriately be managed within primary care settings (4, 5). The high volume of low-priority visits leads to overcrowding in emergency departments, prolonged wait times, and inefficient use of resources allocated for the care of critically ill patients (6).

In pediatric emergency department visits, parents' perception of urgency often does not align with clinical triage assessments, and their tendency to perceive their children's health condition as more serious triggers unnecessary visits (7, 8). Studies indicate that the causes of unnecessary visits are too complex to be explained solely by a lack of information; factors such as difficulties accessing primary care, limited after-hours options, parents' desire for rapid intervention, and underlying systemic drivers that necessitate emergency department use also play a role (6, 9, 10).

Parents' health literacy and educational level are key factors shaping emergency department use (11). While low health literacy increases unnecessary visits, educational level directly influences parents’ ability to rationally analyze clinical situations and make evidence-based decisions (12, 13). Studies examining the profile of non-emergency visits indicate that parental education plays a decisive role in reducing unnecessary emergency room visits (14, 15). Previous studies have identified parental anxiety, misperception of illness severity, barriers to primary care access, and inadequate knowledge of emergency criteria as key drivers of non-urgent pediatric emergency department utilization (14, 16–18). Therefore, unraveling these multidimensional dynamics that influence parents' decision-making processes and presentation motivations is critical for managing emergency department workload.

Although non-urgent pediatric emergency department use has been studied in various settings, evidence simultaneously examining parental responsiveness to informational messaging, perceived urgency perception, and structural access barriers within a middle-income country context lacking a mandatory primary care referral mechanism remains limited. The present study addresses this gap by integrating both behavioral and systemic determinants of low-acuity emergency department utilization in a high-volume tertiary pediatric emergency department in Türkiye, with the aim of generating population-level evidence to inform clinical practice and health policy.

## 2 Materials and methods

### 2.1 Study design

This study is a cross-sectional descriptive study conducted prospectively during the study period. The study was conducted in the pediatric emergency department of a tertiary-level healthcare facility that receives an average of 120,000 pediatric emergency department visits per year, between February 2026 and April 2026.

### 2.2 Participants and eligibility criteria

A formal *a priori* sample size calculation was not performed. Instead, a time-window consecutive sampling strategy was used. To accommodate the institutional workload and availability of the research team, data collection was conducted during designated shifts spanning morning, afternoon, evening, and weekend periods across the study period, during which the primary investigators were physically present in the triage area. This approach was designed to capture the full temporal variation in emergency department attendance patterns, including weekday and weekend presentations across different times of day.

Parents or legal guardians accompanying a child aged 0-18 years who presented to the pediatric emergency department during the active data-collection periods and were triaged into the Blue, Green, or Yellow categories of the Manchester Triage System (MTS) were eligible for inclusion. Individuals were included if they met all predefined eligibility criteria, agreed to participate, and provided sufficiently complete questionnaire data that could be matched with the corresponding hospital records. Parents or legal guardians with insufficient Turkish proficiency to provide informed consent or complete the questionnaire, and those who declined to participate, were not approached for screening, as eligibility for initial contact required basic communicative capacity and willingness to participate. During the active data-collection periods, all remaining potentially eligible parents or legal guardians were consecutively assessed for eligibility.

### 2.3 Triage classification and operational definitions

Triage acuity was assigned by trained emergency department personnel using the Manchester Triage System (MTS). Patients classified in the Red or Orange categories were excluded because the study was designed to examine lower-acuity pediatric emergency department utilization, enrollment could potentially interfere with the provision of immediate or very urgent clinical care. In the study institution, patients assigned to the Green and Blue categories were routed through the same electronic queue-management system and managed within the same lower-acuity care pathway. Accordingly, these categories were combined *a priori* into a study-specific operational group termed the “lower-acuity/non-urgent group.” Patients assigned to the Yellow category constituted the “urgent group.” This grouping was used solely for the purposes of the present analysis and does not modify the original Manchester Triage System classification.

### 2.4 Data collection tools and process

Research data were collected through a structured questionnaire developed by the researchers from a literature review, using face-to-face interviews in the triage area during the child's visit. A pilot study with 10 parents tested item clarity and feasibility, after which the final form was produced. The questionnaire captured parental demographics (age, sex, and educational level), the parents' subjective perception of the child's clinical urgency, motivations for seeking emergency care, and healthcare-access parameters. After the approximately five-minute interview, the child's age, sex, symptom duration, MTS triage code, definitive diagnosis, admission status, readmission, and laboratory test requests were extracted from the HIMS with parental consent and matched to the survey data (19).

The frequency-of-emergency-visits category labeled “more frequent” was operationally defined as more than one emergency department presentation per week. A “test request” was defined as the ordering of any laboratory investigation, including blood or urine tests, or any radiological investigation, including plain radiography or ultrasonography, during the child's emergency department stay.

#### 2.4.1 Parental urgency perception

Parents classified their child's condition as urgent, not urgent, or not sure. For the secondary agreement analysis, “not urgent” and “not sure” responses were combined because neither represented an affirmative parental judgment of urgency, yielding a dichotomous variable of urgent vs. not urgent/not sure.

#### 2.4.2 Clinical-scenario responses (descriptive)

Parents indicated, for each of seven clinical scenarios (sudden-onset dyspnea; chest pain, abdominal pain; high fever, loss of consciousness after a traffic accident; a two-day non-resolving cough; and sudden hemiparesis and speech disturbance), whether they considered it an emergency. Because the study protocol did not include a pre-specified answer key and several scenarios (e.g., chest pain, high fever, and abdominal pain) are clinically ambiguous without information on age, severity, and accompanying findings, these items are reported descriptively as the proportion of parents endorsing each scenario as an emergency. They are not scored as correct or incorrect and are not combined into a knowledge or awareness scale.

#### 2.4.3 Informational-message response

Parents reported their exposure to and perceived usefulness of informational messages about appropriate emergency department use in four categories: “creates awareness,” “not sufficient,” “heard but disregarded,” and “never heard.” For regression, this was dichotomized as an affirmative perception that emergency-use messages create awareness (yes vs. all other responses). This variable reflects a self-reported response to informational messaging and is interpreted strictly as such, not as objective emergency knowledge.

### 2.5 Questionnaire development and reliability

The questionnaire was developed by the research team following a review of the relevant literature and was not previously validated. The structured instrument is therefore described throughout this manuscript as a researcher-developed questionnaire. A pilot study with 10 parents tested item clarity and feasibility; items were refined accordingly. Clinical-scenario items: seven clinical scenarios of varying urgency were included to describe the proportion of parents who endorsed each as an emergency. No pre-specified answer key was defined for these items; they are therefore reported solely as endorsement rates and are not combined into an awareness or knowledge score. Internal consistency of the seven scenario-based items was calculated exploratorily using Cronbach's alpha (*α* = 0.676); however, because the scenarios were not designed as a unidimensional scale and were not combined into a total score, this coefficient is not interpreted as evidence of scale validity.

### 2.6 Statistical analysis

Data analysis was performed using IBM SPSS Statistics version 26.0 (IBM Corp., Armonk, NY, USA). Children classified in the high-acuity Red or Orange Manchester Triage System (MTS) categories were excluded because approaching their parents or legal guardians could interfere with the provision of immediate or very urgent clinical care. In accordance with the pre-specified study protocol, the Blue category was combined with the Green category for analysis, given that both represent lower-acuity presentations managed through the same clinical pathway in the study institution. This decision was made before data collection and did not depend on the observed category counts. The combined analytical “Green/Blue” group comprised all children originally classified as either Green or Blue, resulting in a binary analytical framework consisting of the combined Green/Blue and Yellow categories.

Categorical variables are presented as frequencies and percentages. Continuous variables are presented as mean ± standard deviation or median with interquartile range, as appropriate. Distributional characteristics were assessed using the Kolmogorov–Smirnov test together with visual inspection of histograms and Q-Q plots. Associations between categorical variables were evaluated using Pearson's chi-square test, and effect sizes were reported using Cramér's V without qualitative classification. For bivariate analyses, symptom duration was dichotomized as ≤24 h vs. >24 h.

In the secondary agreement analysis, parental urgency perception was dichotomized as urgent vs. not urgent/unsure. Agreement between dichotomized parental urgency perception and the retained analytical triage categories was assessed using Cohen's kappa with a 95% confidence interval. Because of the skewed marginal distributions, the prevalence-adjusted bias-adjusted kappa (PABAK) was also calculated as 2Po– 1, where Po represents the observed proportion of agreement. Because children in the Red and Orange high-acuity categories were excluded at enrollment, the available triage range was restricted to the combined Green/Blue and Yellow categories. This restriction may mechanically reduce the achievable level of agreement; therefore, the kappa analysis was considered secondary and was interpreted cautiously.

The Mann–Whitney *U*-test and Kruskal–Wallis test were used for comparisons of non-normally distributed continuous or ordinal variables between groups, as appropriate.

Factors associated with an affirmative informational-message response were evaluated using multivariable logistic regression. The model included parental age, parental sex, educational level, and frequency of emergency department visits. Frequency of emergency department visits was entered as a nominal categorical predictor (three dummy variables, with once-yearly use as the reference category) because the categories represented unequal frequency intervals and equal spacing between categories could not be assumed. The model, therefore, comprised six predictor parameters in total. The number of outcome events is reported in the Results; this yielded approximately 20 outcome events per predictor parameter, meeting the conventional ≥10 EPV threshold. Adjusted odds ratios (ORs) with 95% confidence intervals were reported. Model performance was assessed using the likelihood-ratio test and Nagelkerke R^2^ for overall fit, the area under the receiver operating characteristic curve (AUC) for discrimination, and the Hosmer-Lemeshow test for calibration. Educational level was entered as an ordinal predictor, and the assumption of a linear effect was evaluated by comparing the ordinal specification with a categorical (dummy-coded) specification; the likelihood-ratio chi-square difference between the two specifications was non-significant (*p* = 0.74), supporting retention of the ordinal parameterization.

To account for multiple comparisons, Benjamini-Hochberg false-discovery-rate-adjusted *p*-values were reported alongside the raw *p*-values across the 12 bivariate analyses presented in Table 4, using the unrounded raw *p*-values. Statistical inference for these comparisons was based primarily on the adjusted *p*-values. For all other analyses, a two-sided *p*-value < 0.05 was considered statistically significant. Expected cell counts were examined for all contingency tables. When more than 20% of cells had expected counts below five, the Pearson chi-square findings were considered exploratory and were interpreted cautiously.

This study was reported in accordance with the Strengthening the Reporting of Observational Studies in Epidemiology (STROBE) guidelines for cross-sectional studies.

## 3 Ethics approval and consent to participate

The study was approved by the Ethics Committee of the tertiary healthcare institution where the research was conducted (Date: February 25, 2026, Decision Number: E26-802) prior to the initiation of data collection. The research was performed in accordance with the principles of the Declaration of Helsinki. Informed verbal and written consent were obtained from all participating parents/guardians before their inclusion in the study.

## 4 Results

### 4.1 Flowchart of the Study

**Figure 1:**
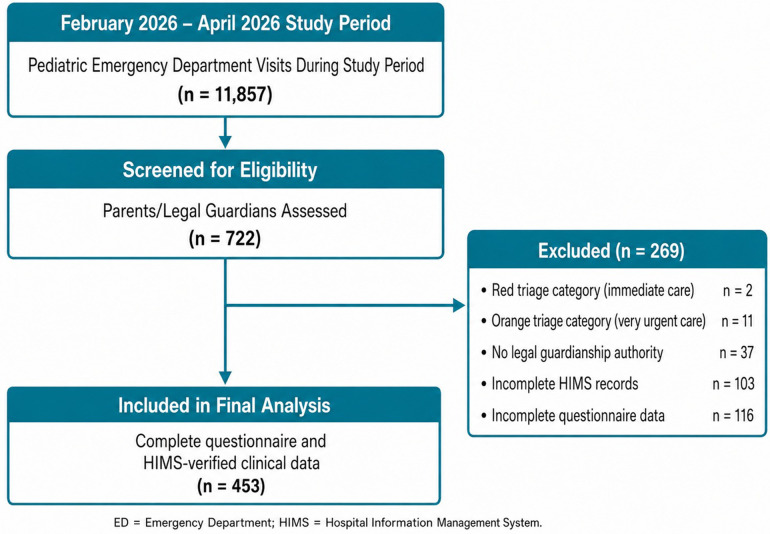
Flowchart of participant screening, exclusion, and final sample selection (*N* = 453) for a pediatric emergency department utilization study at a tertiary center in Türkiye (February-April 2026).

Of the 11,857 pediatric emergency department visits recorded between February 2026 and April 2026, 722 parents or legal guardians were screened for eligibility. After removal of high-acuity triage cases (*n* = 13), participants without legal guardianship authority (*n* = 37), incomplete HIMS records (*n* = 103), and incomplete questionnaire data (*n* = 116), a total of 269 participants were excluded. The final analytical sample comprised 453 participants with complete data (Figure 1).

### 4.2 Sociodemographic and clinical characteristics of the study population

**Figure 2:**
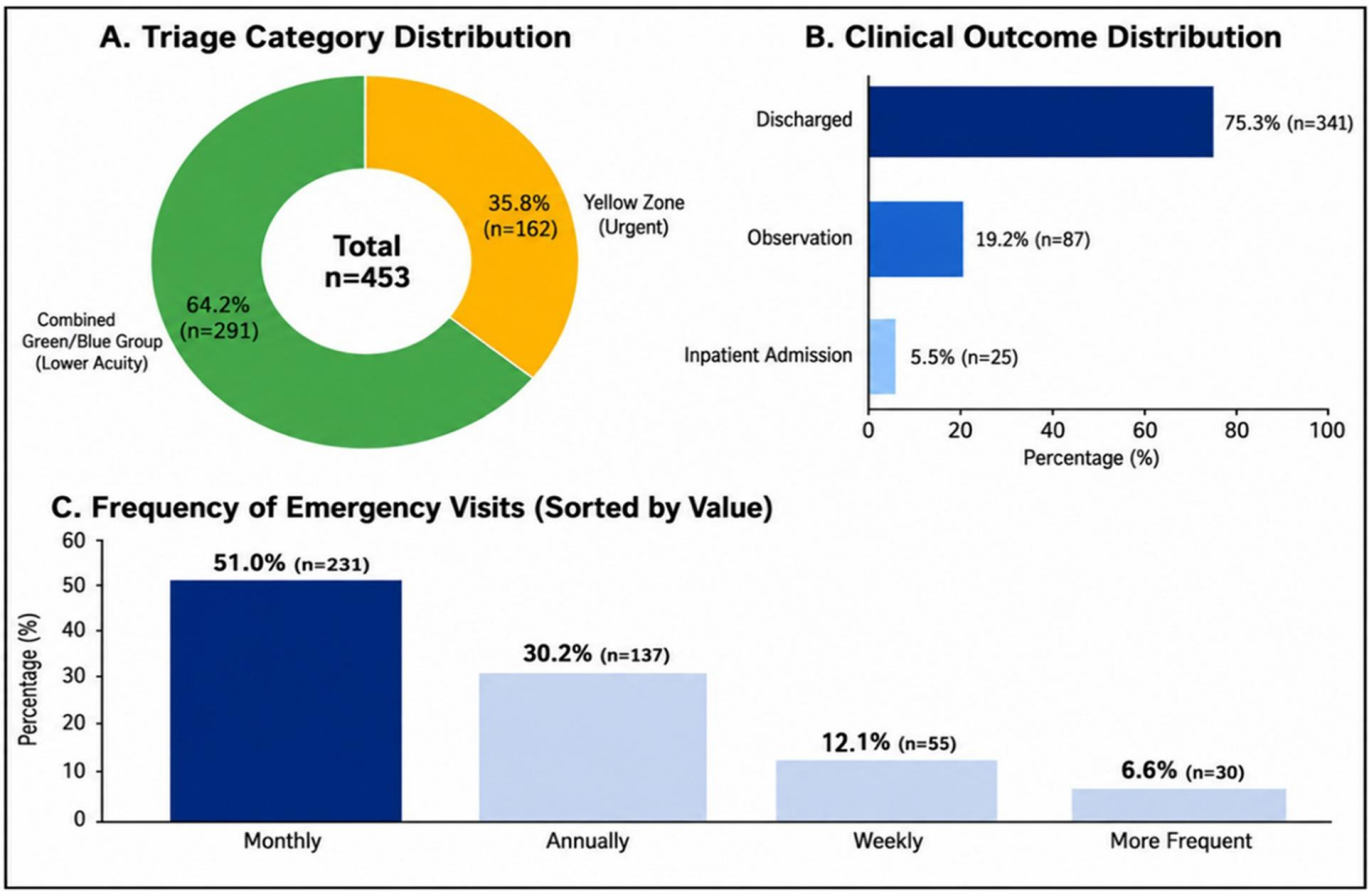
Distribution of **(A)** Manchester triage system categories, **(B)** clinical outcomes (discharge, observation, inpatient admission), and **(C)** self-reported frequency of emergency department visits among 453 pediatric patients at a tertiary-level pediatric emergency department in Türkiye, February-April 2026.

The mean age of the 453 parents was 36.3 ± 7.5 years (median 36.0; IQR 31.0-41.0); most were women (68.2%) and elementary-school graduates (45.3%). The median patient age was 84.0 months (IQR 36.0-132.0). Lower-acuity (combined Green/Blue) presentations comprised 64.2% of cases (*n* = 291), and the most common reported visit frequency was once a month (51.0%). Overall, 75.3% of visits resulted in discharge after examination, 19.2% in observation, and 5.5% in inpatient admission (Table 1; Figure 2). Respiratory tract infections (61.8%) were the leading diagnosis; the most frequently reported symptoms were fever (34.7%) and respiratory distress (27.2%).

**Table 1:** Baseline sociodemographic and clinical characteristics of the study population attending a tertiary-level pediatric emergency department in Türkiye (*N* = 453), February-April 2026.

Variable	Category	Number (n)	Percentage (%)
Parent sex	Female	309	68.2
	Male	144	31.8
Parent age (years)	Mean ± SD (min–max)/median	36.3 ± 7.5 (18–61)/36.0 (31.0–41.0)	
Parent age group	18–29 years	84	18.5
	30–39 years	233	51.4
	40 + years	136	30.0
Educational level	No formal schooling	33	7.3
	Elementary school	205	45.3
	High school	126	27.8
	University	89	19.6
Patient sex	Female	211	46.6
	Male	242	53.4
Patient age (months)	Mean ± SD (min–max)/median	87.8 ± 59.2 (1–204)/84.0 (36.0–132.0)	
Patient age group	< 1 year (infants)	23	5.1
	1–5 years (preschool)	159	35.1
	6–11 years (school age)	168	37.1
	12–17 years (adolescents)	103	22.7
Triage category	Green/Blue (lower acuity)	291	64.2
	Yellow	162	35.8
Frequency of emergency visits	More frequent	30	6.6
	Once a week	55	12.1
	Once a month	231	51.0
	Once a year	137	30.2
Admission status	Discharged	341	75.3
	Observation	87	19.2
	Inpatient admission	25	5.5
Presenting complaints (Multiple responses; the sum of percentages may exceed 100%)	Fever	157	34.7
	Respiratory distress	123	27.2
	Vomiting	109	24.1
	Abdominal pain	94	20.8
	Chest pain	49	10.8
	Inability to eat	39	8.6
	Other*	40	8.8
Presenting diagnosis	Respiratory tract infections	280	61.8
	Acute gastroenteritis	92	20.3
	Asthma attack/Allergic diseases	44	9.7
	Musculoskeletal disorder	15	3.3
	Neurological/Nervous system disorder	11	2.4
	Other*	11	2.4

* Urinary tract infection (*n* = 5), poisoning (*n* = 3), cardiovascular conditions (*n* = 1), bleeding (*n* = 1), and foreign body (*n* = 1) were combined into “Other” to avoid sparse cells. The analytical Green/Blue lower-acuity group comprised 291 patients originally classified as either Green or Blue (per the pre-specified study protocol). These categories were combined *a priori* based on their shared lower-acuity status and identical clinical management pathway; this decision was made before data collection and was independent of the observed category counts.

### 4.3 Reasons for emergency department attendance

Although 66.7% of parents described their child's condition as “urgent,” dichotomized urgency perception was not significantly associated with the retained triage categories (Pearson *χ*^2^ = 1.081, df = 1, *p* = 0.298; Cramér's V = 0.049; Table 2). Agreement between parental urgency perception and MTS triage was minimal (Cohen's *κ* = 0.040, 95% CI −0.035 to 0.116; observed agreement = 47.5%; PABAK = −0.051). This finding should be interpreted cautiously because parental perception was originally measured using three categories, whereas the analytic triage range was restricted to the combined Green/Blue and Yellow categories.

**Table 2:** Concordance analysis between dichotomized subjective parental urgency perception and objective medical triage categories determined by the Manchester triage system (*N* = 453).

Parental perception at presentation	Yellow zone	Green/Blue zone (lower acuity)	Total	*p*-value*
	n (%)	n (%)	n (%)	
Urgent	113 (37.4)	189 (62.6)	302 (100.0)	0.298
Not urgent/Unsure	49 (32.5)	102 (67.5)	151 (100.0)	
Total	162 (35.8)	291 (64.2)	453 (100.0)	

* Uncorrected Pearson chi-square: *χ*^2^ = 1.081, df = 1, *p* = 0.298; Cramér's V = 0.049. Cohen's *κ* = 0.040 (95% CI −0.035 to 0.116); PABAK = −0.051.

### 4.4 Motivations for emergency department utilization

The distribution of parents' reasons for choosing the emergency department did not differ significantly across triage categories (*χ*^2^ = 4.854, df = 4, *p* = 0.303; Table 3). Independent of triage category, the most frequently reported reason was the expectation of “faster examination,” expressed by 36.1% of parents (*n* = 162). Regarding healthcare-system preferences, 53.4% of parents (*n* = 242) indicated they might use after-hours outpatient services as an alternative, while 46.6% (*n* = 211) would still use the emergency department in similar situations.

**Table 3:** Self-reported reasons for emergency department attendance among 449 parents, stratified by triage acuity (lower-acuity green/blue vs. urgent Yellow).

Reason for choosing the ED	Green/Blue zone (lower acuity) n (%)	Yellow zone n (%)	Total n (%)	*p*-value
Desire for a faster examination	106 (65.4)	56 (34.6)	162 (36.1)	0.303
My child's condition is urgent	65 (57.0)	49 (43.0)	114 (25.4)	
Unable to get an outpatient appointment	60 (67.4)	29 (32.6)	89 (19.8)	
No place to go outside of working hours	42 (72.4)	16 (27.6)	58 (12.9)	
Habit	16 (61.5)	10 (38.5)	26 (5.8)	
Total	289 (64.4)	160 (35.6)	449 (100.0)	

Row percentages are shown. Four participants did not provide a valid response to this item; therefore, the analysis was based on 449 participants. Pearson chi-square (*χ*^2^ = 4.854, df = 4, *p* = 0.303).

### 4.5 Clinical outcomes and resource utilization by triage category

Among lower-acuity (Green/Blue) presentations (*n* = 291), 38.1% (111/291) underwent laboratory or radiological investigations, and 7.9% (23/291) resulted in observation or inpatient admission (19 in observation and 4 admitted to the ward). For comparison, the corresponding whole-cohort rates were 53.0% (240/453) for investigations and 24.7% (112/453) for observation or admission; these are reported as cohort-wide figures.

An association was observed between triage category and admission status (*χ*^2^ = 123.987, df = 2, *p* < 0.001; Cramér's V = 0.523). Associations were also found between presenting diagnosis and triage category (*p* < 0.001) and between triage and test request (*p* < 0.001). Owing to sparse cells (7 of 18 cells, 38.9%, with expected counts below five), the association between presenting diagnosis and admission status was evaluated using the Fisher-Freeman-Halton exact test; the Pearson chi-square statistic (*χ*^2^ = 70.02, df = 10) is presented in Table 4 for comparability, and statistical inference is based on the exact *p*-value (*p* < 0.001). This result should be regarded as exploratory. The patient age group  ×  admission status association, computed using the consistent four-category age grouping, was *χ*^2^ = 85.77, df = 6, *p* < 0.001 (Cramér's V = 0.308). No significant association was found between symptom duration and parental urgency perception (*χ*^2^ = 0.043, df = 1, *p* = 0.835; Cramér's V = 0.010). In the presenting diagnosis  ×  triage analysis, 2 of 12 cells (16.7%) had expected counts below five, which falls below the 20% exploratory threshold and therefore does not require cautious interpretation. In the presenting diagnosis×admission analysis, 7 of 18 cells (38.9%) had expected counts below five, exceeding this threshold; therefore, the latter finding should be regarded as exploratory and interpreted cautiously.

**Table 4:** Bivariate associations among sociodemographic, clinical, and utilization variables (Pearson chi-square, cramér's V, and Benjamini–hochberg-adjusted *p*-values).

Variable pair	*χ* ^2^	df	*p* (raw)	*p* (BH-adj.)	Cramér's V
Triage×Admission status	123.99	2	<0.001	<0.001	0.523
Presenting diagnosis×Triage	65.62	5	<0.001	<0.001	0.381
Presenting diagnosis×Admission	70.02	10	<0.001 (exact)	<0.001	0.278
Patient age group×Admission	85.77	6	<0.001	<0.001	0.308
Triage×Test request	71.90	1	<0.001	<0.001	0.398
Education level×Message response	11.38	3	0.010	0.017	0.158
Perceived urgency×Message response	7.35	1	0.007	0.013	0.127
Patient sex×Admission	5.20	2	0.074	0.099	0.107
ED-visit frequency×Message response	7.95	3	0.047	0.071	0.132
Perceived urgency×Triage	1.08	1	0.298	0.358	0.049
Symptom duration×Perceived urgency	0.04	1	0.835	0.836	0.010
Parent sex×Message response	0.18	1	0.672	0.732	0.020

Uncorrected Pearson chi-square tests; *p* (BH-adj.) = Benjamini-Hochberg false-discovery-rate adjustment applied across the 12 tests using unrounded *p*-values. “Message response” denotes the affirmative informational-message response (Question 10). For presenting diagnosis×triage, 2 of 12 cells (16.7%) had expected counts below five, which falls below the 20% threshold for exploratory classification; for presenting diagnosis×admission, 7 of 18 cells (38.9%) had expected counts below five, exceeding this threshold. The latter result is therefore considered exploratory and should be interpreted cautiously.

### 4.6 Parental appraisal and risk perception of clinical scenarios

The proportion of parents endorsing each scenario as an emergency varied widely (Table 5): loss of consciousness after a traffic accident, 53.0%; chest pain, 43.3%; sudden hemiparesis and speech disturbance, 42.4%; sudden-onset dyspnea, 40.4%; high fever, 38.4%; abdominal pain, 19.6%; and a two-day non-resolving cough, 13.7%. These are reported as endorsement rates (“considered an emergency”), not as correct-answer rates, because no pre-specified answer key was defined.

**Table 5:** Proportion of parents endorsing each of seven standardized clinical scenarios as a medical emergency.

Clinical scenario	“Considered an emergency” n (%)
Loss of consciousness after a traffic accident	240 (53.0)
Chest pain	196 (43.3)
Sudden hemiparesis and speech disturbance	192 (42.4)
Sudden-onset dyspnea	183 (40.4)
High fever	174 (38.4)
Abdominal pain	89 (19.6)
Two-day non-resolving cough	62 (13.7)

### 4.7 Predictors of responsiveness to emergency-use informational messaging

**Figure 3:**
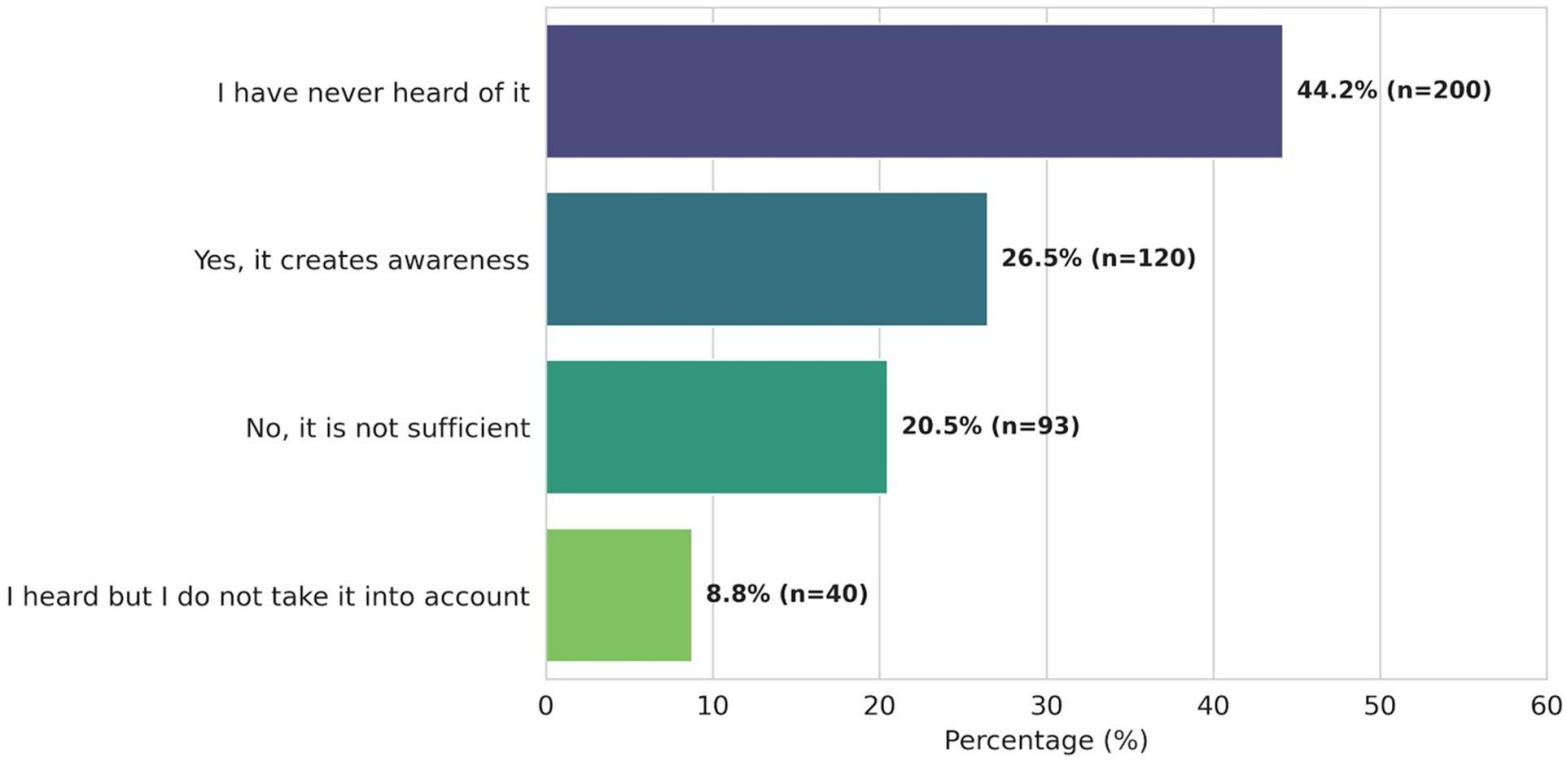
Parental responsiveness to public health informational messaging on emergency department use among 453 parents attending a tertiary-level pediatric emergency department in Türkiye, February-April 2026.

When asked whether they had previously encountered informational messages regarding emergency department use, 44.2% (*n* = 200) reported never having heard such information (Figure 3). The proportion reporting that informational messages created awareness increased descriptively from 18.2% among parents without formal schooling to 37.1% among university graduates. The association between educational level and affirmative informational-message response was statistically significant in the bivariate analysis (*χ*^2^ = 11.38, df = 3, raw *p* = 0.010; BH-adjusted *p* = 0.017). There were 120 affirmative outcome events across six predictor parameters. In multivariable logistic regression, educational level remained independently associated with an affirmative informational-message response (Table 6): each one-level increase was associated with 1.45-fold higher odds (adjusted OR = 1.45; 95% CI 1.14-1.86; *p* = 0.003).

**Table 6:** Multivariable logistic regression analysis of sociodemographic and behavioral predictors of affirmative response to informational messages on emergency department use (*N* = 453).

Predictor	B (SE)	Adjusted OR	95% CI	*p*-value
Parent age (per year)	−0.000 (0.015)	1.00	0.97–1.03	0.974
Female sex (reference: male)	−0.022 (0.238)	0.98	0.61–1.56	0.927
Educational level (per category)	0.374 (0.124)	1.45	1.14–1.86	0.003
ED visits: once a week	−0.881 (0.411)	0.41	0.19–0.93	0.032
ED visits: once a month	−0.298 (0.239)	0.74	0.46–1.19	0.212
ED visits: more frequent	−0.856 (0.528)	0.42	0.15–1.20	0.105

Outcome: affirmative response that informational messages created awareness (yes vs. all other responses). B = logit coefficient; SE = standard error; OR = odds ratio; CI = confidence interval. Once-yearly ED use was the reference category for visit frequency. Overall Wald test for ED-visit frequency: χ^2^ = 6.35, df = 3, *p* = 0.096. Model likelihood-ratio χ^2^ = 17.57, df = 6, *p* = 0.007; Nagelkerke R^2^ = 0.056; AUC = 0.620; Hosmer-Lemeshow χ^2^ = 4.17, df = 8, *p* = 0.842. There were 120 outcome events and six predictor parameters. Comparison of the ordinal educational-level specification with a categorical (dummy-coded) specification yielded a non-significant likelihood-ratio chi-square difference (*p* = 0.74), supporting retention of the ordinal parameterization.

## 5 Discussion

This study showed that a substantial proportion of pediatric emergency department visits is shaped by factors beyond objective clinical urgency. Nearly two-thirds of visits (64.2%) were classified in the lower-acuity (Green/Blue) group, and the agreement between parental urgency perception and objective triage was minimal (Cohen's *κ*=0.040). Educational level was independently associated with parents' responsiveness to informational messaging; however, this association was not accompanied by closer alignment between parental urgency perception and objective triage, as discussed below.

### 5.1 Low-acuity utilization and parental urgency perception

Overcrowding in emergency departments is a recognized global health problem. Sartini et al. (20) noted that overcrowding increases both patients' wait times and staff workload, contributes to burnout, disrupts patient follow-up and care quality, and threatens patient safety by delaying treatment for those requiring urgent intervention. A systematic review of 20 US studies found that 37% of pediatric emergency department visits (up to 58% in some states) were non-urgent (21), while an Australian review reported an average of 41% (2). A Turkish study found that 81.1% of visits were non-emergency (5). Within our selected analytic cohort, the 64.2% of presentations classified as lower-acuity (Green/Blue) highlight a notable concentration of low-acuity presentations. However, because high-acuity presentations were excluded at recruitment, and data collection did not encompass the entirety of total PED attendances, this proportion represents the baseline of our analyzed subset and should not be interpreted as the absolute prevalence of low-acuity visits across the entire department.

### 5.2 Educational level and informational-message responsiveness

Even when classified as low-priority, non-emergency visits consume significant resources through diagnostic testing and treatment (22). Consistent with prior evidence linking parental education to specialist choice and primary care utilization, higher educational level in our study was independently associated with greater informational-message responsiveness (adjusted OR = 1.45; 95% CI 1.14-1.86; *p* = 0.003) (12, 13). Notably, this association was not accompanied by closer alignment between perceived and objective urgency, suggesting that informational responsiveness does not necessarily translate into appropriate care-seeking behavior. The observation that investigations were requested in 38.1% (111/291) of lower-acuity presentations further illustrates the resource implications of low-acuity emergency department use. Taken together, these findings highlight a critical distinction between informational responsiveness and clinical decision-making. Higher educational attainment was associated with greater receptiveness to emergency-use messaging but was not accompanied by improved agreement between parental urgency perception and objective triage classification (Cohen's *κ*=0.040; PABAK = –0.051). This dissociation suggests that educational attainment may enhance awareness of health messages without meaningfully altering risk appraisal or healthcare-seeking behavior under conditions of clinical uncertainty. When parents perceive a potentially serious illness, emotional factors and the fear of missing a critical diagnosis may override factual knowledge, a pattern consistent with the broader literature on parental risk tolerance in pediatric emergencies (23, 24). These findings imply that interventions focusing exclusively on information provision are unlikely to produce substantial reductions in non-urgent pediatric emergency department utilization, and that behavioral and structural approaches may be necessary complements.

### 5.3 Structural access factors and preference for emergency care

Low rates of primary care utilization prior to emergency department visits have been documented in the Turkish context (25), a pattern McLauchlan et al. (26) attributed to parental clinical uncertainty and low risk tolerance, with parents viewing resource-rich emergency departments as the safest option and bypassing primary care entirely following unsatisfactory referral experiences. Consistent with these findings, our data suggest that frequent emergency department use coexists with low primary-care utilization, reflecting systemic preferences rather than information deficits alone, a pattern pointing to the need for structural reform in the family-medicine referral pathway (12, 26, 27).

Parental preference for emergency departments over primary care likely reflects a convergence of individual perceptions and systemic barriers. Calicchio et al. (16) reported that nearly half of parents (48.4%) chose the emergency department expecting faster, more reliable care, while Marbella et al. (28) identified perceived access delays, parental anxiety, and the perception of the emergency department as the sole provider of definitive solutions as key decision-making drivers. Our findings are consistent with this pattern, suggesting that emergency department attendance among low-acuity cases is partly driven by the pursuit of rapid evaluation rather than perceived clinical necessity.

Certain symptoms appear to exert a strong alarm effect on parental urgency perception. Prior studies have shown that acute respiratory distress, repeated vomiting, and fever are frequently interpreted as indicators of serious harm irrespective of actual clinical severity (9, 13, 29). In our study, 64.0% of cases presenting with respiratory distress, 66.9% of those with fever, and 59.0% of those with vomiting or feeding difficulties were classified in the lower-acuity group, demonstrating a systematic discrepancy between symptom-driven parental urgency appraisal and objective triage. Although parental self-assessment partially aligns with clinical urgency, this concordance remains limited; parental anxiety is highly prevalent in pediatric emergency settings (24, 30), and Pehlivanturk-Kizilkan et al. (23) showed that overestimation of emergency severity is associated with younger parental age and lower educational attainment, suggesting that anxiety-driven risk appraisal may override objective clinical judgment.

### 5.4 Policy implications

The high volume of pediatric emergency department visits cannot be explained solely by parental knowledge deficits; structural features of the healthcare system appear to contribute independently. The finding that 53.4% of parents would consider after-hours outpatient services suggests unmet demand for accessible primary care alternatives, though objective access barriers were not directly measured. Hong et al. (31) demonstrated that improving after-hours primary care access is associated with reductions in emergency department utilization ranging from 2% to 49% globally a finding particularly relevant to Türkiye, where the absence of a mandatory referral mechanism allows unrestricted direct access to tertiary emergency services. Addressing this structural gap may represent a more sustainable strategy for reducing low-acuity emergency department burden than public education initiatives alone (31).

## 6 Strengths and limitations

The primary strength of this study is that it comprehensively examined a common problem in pediatric emergency medicine practice using a large sample of 453 participants and advanced multivariate statistical analysis methods. However, certain limitations should be considered when interpreting the findings. The study's single-center, cross-sectional design limits the generalizability of the results and does not allow for the establishment of causal relationships between variables. In addition, the multivariable logistic regression model demonstrated limited discriminative ability (AUC = 0.620) and explained only a modest proportion of the variance (Nagelkerke R^2^ = 0.056), indicating that determinants beyond those measured contribute to informational-message responsiveness; the model should therefore be regarded as exploratory. Additionally, the collection of data based on self-report increases the risk of response bias due to the social desirability effect. A limitation of our study is that the reasons why parents do not prefer primary care services (e.g., lack of trust, distance, communication with the physician, etc.) were not explored in depth or directly. Future studies focusing on these motivations would be beneficial for analyzing the systemic burden.

## 7 Conclusion

This study demonstrates that a significant proportion of pediatric emergency department visits is shaped not only by objective clinical urgency but also by parents' subjective risk appraisal, responsiveness to health information, and expectations of rapid access to care. Parental educational level was the strongest independent correlate of receptiveness to emergency-use informational messaging; however, this association was not accompanied by improved alignment between parental urgency perception and objective triage classification, indicating that informational responsiveness and clinical decision-making represent distinct constructs.

These findings indicate that low-acuity pediatric emergency department utilization cannot be attributed solely to parental knowledge deficits but reflects a more complex interaction between individual risk perception and structural characteristics of the healthcare system. Educational campaigns alone are therefore unlikely to produce substantial reductions in non-urgent utilization.

Future strategies should integrate public education with structural reforms in primary care, particularly the expansion of accessible after-hours services. Critically, the absence of a mandatory referral mechanism in Türkiye's healthcare system, which allows unrestricted direct access to tertiary emergency departments without prior primary care consultation, represents a key structural enabler of non-urgent utilization that must be addressed through policy reform. Combining improved after-hours primary care accessibility with a structured referral pathway could provide credible alternatives to emergency department attendance and contribute to the sustainable management of pediatric emergency department overcrowding.

## Data Availability

The raw data supporting the conclusions of this article will be made available by the authors, without undue reservation.
