# Supplementary material for: Beyond clinical urgency: parental awareness, educational level, and non-urgent pediatric emergency department utilization
Source: Front Pediatr. 2026 Jul 22;14:1890076. doi: 10.3389/fped.2026.1890076 (PMC13437803; doi:10.3389/fped.2026.1890076)
Supplement: Supplementary file 1 [file Table1.docx]

STROBE Statement—checklist of items that should be included in reports of observational studies

|  | Item No. | Recommendation | Page  No. | Relevant text from manuscript |
| --- | --- | --- | --- | --- |
| **Title and abstract** | 1 | (*a*) Indicate the study’s design with a commonly used term in the title or the abstract | 1 | Title: 'Parental Urgency Perception, Educational Level, and Healthcare-Seeking Reasons Among Pediatric Emergency Department Attendees: A Cross-Sectional Study'. Abstract: 'This cross-sectional descriptive study, conducted prospectively…' |
|  |  | (*b*) Provide in the abstract an informative and balanced summary of what was done and what was found | 1 | Abstract provides a structured summary of design, setting, participants (n=453), main outcomes (kappa=0.040; adjusted OR=1.45), and conclusions regarding structural access barriers. |
| Introduction | | | |  |
| Background/rationale | 2 | Explain the scientific background and rationale for the investigation being reported | 2 | Introduction: 'Approximately 40% to 80% of pediatric emergency department presentations involve non-urgent clinical conditions… High volumes of low-priority visits contribute to overcrowding, prolonged wait times, and inefficient use of resources…' |
| Objectives | 3 | State specific objectives, including any prespecified hypotheses | 2 | '…we aimed to systematically assess parents' emergency department use, their reasons for seeking care, and their perceptions of urgency, and to inform preventive and improvement strategies for more effective, appropriate, and sustainable use of pediatric emergency departments.' |
| Methods | | | |  |
| Study design | 4 | Present key elements of study design early in the paper | 2 | Methods – Study Design: 'This cross-sectional descriptive study was conducted prospectively between February 26 and April 30, 2026, at a tertiary pediatric emergency department managing approximately 120,000 annual visits.' |
| Setting | 5 | Describe the setting, locations, and relevant dates, including periods of recruitment, exposure, follow-up, and data collection | 2 | Methods – Study Design: 'This cross-sectional descriptive study was conducted prospectively between February 26 and April 30, 2026, at a tertiary pediatric emergency department managing approximately 120,000 annual visits. Data collection was restricted to prespecified 8-hour shifts covering alternating morning, evening, and weekend periods (February 26–April 30, 2026). Institutional ethics approval was granted on February 25, 2026.' |
| Participants | 6 | (*a*) *Cohort study*—Give the eligibility criteria, and the sources and methods of selection of participants. Describe methods of follow-up  *Case-control study*—Give the eligibility criteria, and the sources and methods of case ascertainment and control selection. Give the rationale for the choice of cases and controls  *Cross-sectional study*—Give the eligibility criteria, and the sources and methods of selection of participants | 2–3 | Results: Primary outcome – affirmative informational-message response: 120/453 (26.5%) reported that informational messages created awareness. Secondary outcome – parental urgency perception: 302/453 (66.7%) classified their child’s condition as urgent. Triage distribution: 291 (64.2%) lower-acuity Green/Blue, 162 (35.8%) Yellow. Among lower-acuity presentations: 38.1% (111/291) underwent investigations; 7.9% (23/291) resulted in observation or inpatient admission. |
|  |  | (*b*) *Cohort study*—For matched studies, give matching criteria and number of exposed and unexposed  *Case-control study*—For matched studies, give matching criteria and the number of controls per case |  |  |
| Variables | 7 | Clearly define all outcomes, exposures, predictors, potential confounders, and effect modifiers. Give diagnostic criteria, if applicable | 3 | Data Collection: Primary outcome – affirmative informational-message response (dichotomised). Secondary – parental urgency perception (urgent vs not urgent/unsure). Predictor – educational level (ordinal). Covariates – parental age, sex, ED-visit frequency. Clinical triage category (Green/Blue combined vs Yellow) as the objective comparator. |
| Data sources/ measurement | 8* | For each variable of interest, give sources of data and details of methods of assessment (measurement). Describe comparability of assessment methods if there is more than one group | 3 | Data Collection Tools: 'Research data were collected through a structured questionnaire… using face-to-face interviews in the triage area.' Clinical data (triage code, diagnosis, admission, test requests) extracted from the Hospital Information Management System (HIMS) and matched to survey data. |
| Bias | 9 | Describe any efforts to address potential sources of bias | 3 | Sample and Participants: consecutive recruitment during active shifts minimised selection bias. Ethics and Consent section: written informed consent obtained; direct identifiers removed. Limitations acknowledge social-desirability response bias and single-centre design. |
| Study size | 10 | Explain how the study size was arrived at | 2 | Sample and Participants: 'A formal a priori sample size calculation was not performed. Instead, a time-window consecutive sampling strategy was used… The final analytical sample consisted of 453 parents or legal guardians.' Regression EPV: 120 affirmative outcome events across 6 predictor parameters (≈20 outcome events per predictor parameter), meeting the conventional ≥10 EPV threshold. |

Continued on next page

| Quantitative variables | 11 | Explain how quantitative variables were handled in the analyses. If applicable, describe which groupings were chosen and why | 3–4 | Statistical Analysis: 'Distributional characteristics were assessed using the Kolmogorov–Smirnov test together with visual inspection of histograms and Q–Q plots.' Symptom duration dichotomised as ≤24 h vs >24 h. Educational level entered as ordinal predictor; linearity evaluated against categorical specification. ED-visit frequency entered as nominal categorical predictor. |
| --- | --- | --- | --- | --- |
| Statistical methods | 12 | (*a*) Describe all statistical methods, including those used to control for confounding | 3–4 | Statistical Analysis: Pearson chi-square + Cramér's V for categorical associations; Mann–Whitney U and Kruskal–Wallis for non-normal continuous/ordinal variables; Cohen's kappa + PABAK for agreement; multivariable logistic regression (adjusted ORs with 95% CI) for confounding control. |
|  |  | (*b*) Describe any methods used to examine subgroups and interactions | 3–4 | Green-zone subgroup analyses reported separately for investigation rates (38.1%; 111/291) and admission rates (7.9%; 23/291). No formal interaction terms were pre-specified; triage-stratified results presented descriptively in Table 3 and Table 4. |
|  |  | (*c*) Explain how missing data were addressed | 2–3 | Sample and Participants: '30 records were excluded because of incomplete questionnaire data or irreconcilable discrepancies with HIMS records.' Four participants omitted the ED-reason item (Table 3 n=449). No imputation was performed. |
|  |  | (*d*) *Cohort study*—If applicable, explain how loss to follow-up was addressed  *Case-control study*—If applicable, explain how matching of cases and controls was addressed  *Cross-sectional study*—If applicable, describe analytical methods taking account of sampling strategy | 2–3 | Results: Primary outcome – affirmative informational-message response: 120/453 (26.5%) reported that informational messages created awareness. Secondary outcome – parental urgency perception: 302/453 (66.7%) classified their child’s condition as urgent. Triage distribution: 291 (64.2%) lower-acuity Green/Blue, 162 (35.8%) Yellow. Among lower-acuity presentations: 38.1% (111/291) underwent investigations; 7.9% (23/291) resulted in observation or inpatient admission. |
|  |  | (*e*) Describe any sensitivity analyses | 3–4 | Statistical Analysis: Linearity of the educational-level effect evaluated by comparing ordinal vs categorical specification (likelihood-ratio chi-square difference p=0.74). Benjamini–Hochberg FDR adjustment applied across 12 bivariate tests as a sensitivity check on multiple comparisons. |
| Results | | | | |
| Participants | 13* | (a) Report numbers of individuals at each stage of study—eg numbers potentially eligible, examined for eligibility, confirmed eligible, included in the study, completing follow-up, and analysed | 4 | Results / Figure 1: 11,857 total visits during study period; 722 screened during active shifts; 269 excluded (112 high-acuity triage; 64 refused consent; 38 language barrier; 25 no guardianship; 30 incomplete data); 453 analysed. |
|  |  | (b) Give reasons for non-participation at each stage | 2–3 | Sample and Participants: 'Of these, 269 were excluded: 112 children classified in Red/Orange MTS; 64 parents declined informed consent; 38 insufficient Turkish proficiency; 25 no legal guardianship authority; 30 incomplete questionnaire data or HIMS discrepancies.' |
|  |  | (c) Consider use of a flow diagram | 3 | Figure 1: 'Flowchart detailing the study recruitment process' – presented in the manuscript. |
| Descriptive data | 14* | (a) Give characteristics of study participants (eg demographic, clinical, social) and information on exposures and potential confounders | 4 | Table 1: sociodemographic (parent sex, age, education; patient sex, age, age group) and clinical (triage category, visit frequency, admission status, presenting complaints, diagnosis) characteristics reported for all 453 participants. |
|  |  | (b) Indicate number of participants with missing data for each variable of interest | 4 | Results: 4 participants did not respond to the ED-reason item (Table 3, n=449); no other variable had missing data after exclusion of the 30 records with incomplete questionnaires. |
|  |  | (c) *Cohort study*—Summarise follow-up time (eg, average and total amount) |  |  |
| Outcome data | 15* | *Cohort study*—Report numbers of outcome events or summary measures over time |  |  |
|  |  | *Case-control study—*Report numbers in each exposure category, or summary measures of exposure |  |  |
|  |  | *Cross-sectional study—*Report numbers of outcome events or summary measures | 2–3 | Results: Primary outcome – affirmative informational-message response: 120/453 (26.5%) reported that informational messages created awareness. Secondary outcome – parental urgency perception: 302/453 (66.7%) classified their child’s condition as urgent. Triage distribution: 291 (64.2%) lower-acuity Green/Blue, 162 (35.8%) Yellow. Among lower-acuity presentations: 38.1% (111/291) underwent investigations; 7.9% (23/291) resulted in observation or inpatient admission. |
| Main results | 16 | (*a*) Give unadjusted estimates and, if applicable, confounder-adjusted estimates and their precision (eg, 95% confidence interval). Make clear which confounders were adjusted for and why they were included | 5 | Table 6: adjusted OR for educational level=1.45 (95% CI 1.14–1.86; p=0.003). Unadjusted bivariate association: chi-square=11.38, p=0.010 (BH-adj. p=0.017). All covariates (parental age, sex, ED-visit frequency) reported with adjusted ORs and 95% CIs. |
|  |  | (*b*) Report category boundaries when continuous variables were categorized | 3–4 | Statistical Analysis: symptom duration dichotomised as ≤24 h vs >24 h. Educational level categories: no formal schooling / elementary school / high school / university (ordinal). Patient age groups: <1 year / 1–5 years / 6–11 years / 12–17 years. ED-visit frequency: more frequent (>1/week) / once a week / once a month / once a year. |
|  |  | (*c*) If relevant, consider translating estimates of relative risk into absolute risk for a meaningful time period | N/A | Not applicable – the outcome is a self-reported attitudinal variable (informational-message response), not a disease outcome; absolute risk translation is not meaningful in this context. |

Continued on next page

| Other analyses | 17 | Report other analyses done—eg analyses of subgroups and interactions, and sensitivity analyses | 4–5 | Results: green-zone subgroup analyses (investigation and admission rates); ordinal vs categorical specification comparison for educational level (sensitivity analysis); Table 4 presents 12 bivariate associations with FDR-adjusted p-values; Table 5 reports descriptive scenario-endorsement rates. |
| --- | --- | --- | --- | --- |
| Discussion | | | | |
| Key results | 18 | Summarise key results with reference to study objectives | 5–6 | Discussion: 'This study showed that a substantial proportion of pediatric emergency department visits is shaped by factors other than objective clinical urgency. Nearly two-thirds (64.2%) of presentations were classified as lower-acuity (combined Green/Blue group); agreement between dichotomised parental urgency perception and the two-category analytical triage classification was minimal (Cohen’s κ = 0.040; PABAK = −0.051). Educational level was independently associated with informational-message responsiveness (adjusted OR = 1.45; 95% CI 1.14–1.86; p = 0.003). These represent separate findings.' |
| Limitations | 19 | Discuss limitations of the study, taking into account sources of potential bias or imprecision. Discuss both direction and magnitude of any potential bias | 6 | Strengths and Limitations: single-centre cross-sectional design limits generalisability; self-reported data risk social-desirability bias; informational-message response reflects self-reported responsiveness, not objective knowledge; exclusion of urgent cases truncates the triage range; recruitment limited to prespecified data-collection shifts may have introduced time-of-day or day-of-week selection bias; repeated presentations managed by retaining only the first eligible encounter; AUC = 0.620 indicates limited model discriminative ability; depth of primary-care preference barriers not explored. |
| Interpretation | 20 | Give a cautious overall interpretation of results considering objectives, limitations, multiplicity of analyses, results from similar studies, and other relevant evidence | 6 | Conclusion: 'Parental urgency perception showed minimal agreement with the restricted two-category analytical triage classification, and educational level was independently associated with responsiveness to informational messaging—though not with closer alignment between perceived and objective urgency. These represent separate findings. These findings suggest that awareness-raising initiatives alone are unlikely to be sufficient. Models integrating parental decision support with accessible after-hours primary care may warrant evaluation in future intervention studies.' |
| Generalisability | 21 | Discuss the generalisability (external validity) of the study results | 6 | Strengths and Limitations / Contribution: 'The single-center, cross-sectional design limits generalizability.' Contribution statement notes findings are from a middle-income-country tertiary-care setting without mandatory referral, applicable to comparable healthcare systems. |
| Other information | |  | | |
| Funding | 22 | Give the source of funding and the role of the funders for the present study and, if applicable, for the original study on which the present article is based | 7 | Funding: 'No financial support was received for this study.' Conflicts of interest: 'The authors declare that they have no conflicts of interest.' |

*Give information separately for cases and controls in case-control studies and, if applicable, for exposed and unexposed groups in cohort and cross-sectional studies.

**Note:** An Explanation and Elaboration article discusses each checklist item and gives methodological background and published examples of transparent reporting. The STROBE checklist is best used in conjunction with this article (freely available on the Web sites of PLoS Medicine at http://www.plosmedicine.org/, Annals of Internal Medicine at http://www.annals.org/, and Epidemiology at http://www.epidem.com/). Information on the STROBE Initiative is available at www.strobe-statement.org.
